# Speeding *Cis*-*Trans* Regulation Discovery by Phylogenomic Analyses Coupled with Screenings of an Arrayed Library of Arabidopsis Transcription Factors

**DOI:** 10.1371/journal.pone.0021524

**Published:** 2011-06-27

**Authors:** Gabriel Castrillo, Franziska Turck, Magalie Leveugle, Alain Lecharny, Pilar Carbonero, George Coupland, Javier Paz-Ares, Luis Oñate-Sánchez

**Affiliations:** 1 Department of Plant Molecular Genetics, Centro Nacional de Biotecnología, Consejo Superior de Investigaciones Científicas, Cantoblanco, Madrid, Spain; 2 Department of Plant Developmental Biology, Max Planck Institute for Plant Breeding Research, Cologne, Germany; 3 Unité de Recherche en Génomique Végétale, Institut Scientifique de Recherche Agronomique and Centre National de la Recherche Scientifique, Evry, France; 4 Departamento de Biotecnología and Centro de Biotecnología y Genómica de Plantas, Universidad Politécnica de Madrid, Pozuelo de Alarcón, Madrid, Spain; Instituto de Biología Molecular y Celular de Plantas, Spain

## Abstract

Transcriptional regulation is an important mechanism underlying gene expression and has played a crucial role in evolution. The number, position and interactions between *cis*-elements and transcription factors (TFs) determine the expression pattern of a gene. To identify functionally relevant *cis*-elements in gene promoters, a phylogenetic shadowing approach with a lipase gene (*LIP1*) was used. As a proof of concept, in silico analyses of several *Brassicaceae LIP1* promoters identified a highly conserved sequence (*LIP1* element) that is sufficient to drive strong expression of a reporter gene in planta. A collection of ca. 1,200 *Arabidopsis thaliana* TF open reading frames (ORFs) was arrayed in a 96-well format (RR library) and a convenient mating based yeast one hybrid (Y1H) screening procedure was established. We constructed an episomal plasmid (pTUY1H) to clone the *LIP1* element and used it as bait for Y1H screenings. A novel interaction with an HD-ZIP (AtML1) TF was identified and abolished by a 2 bp mutation in the *LIP1* element. A role of this interaction in transcriptional regulation was confirmed in planta. In addition, we validated our strategy by reproducing the previously reported interaction between a MYB-CC (PHR1) TF, a central regulator of phosphate starvation responses, with a conserved promoter fragment (*IPS1* element) containing its cognate binding sequence. Finally, we established that the *LIP1* and *IPS1* elements were differentially bound by HD-ZIP and MYB-CC family members in agreement with their genetic redundancy in planta. In conclusion, combining in silico analyses of orthologous gene promoters with Y1H screening of the RR library represents a powerful approach to decipher *cis-* and *trans*-regulatory codes.

## Introduction

The control of gene expression is crucial for proper development in any living organism. Transcriptional regulation is an important mechanism underlying gene expression that has been a powerful driving force in the evolution of function and form [1] and is considered to have enormous biotechnological potential in the manipulation of agronomic traits [2 and references therein]. Transcriptional control is mediated by short DNA sequences (*cis*-elements) located in gene promoters that are bound by transcription factors (TFs). Combinatorial control driven by different *cis*-elements and their corresponding TF proteins at a given promoter is an important but not well understood area of plant gene regulation [3]. To reveal the complexity of gene transcriptional regulation it is necessary to identify all functionally relevant regulatory elements (*cis* regulatory code) as well as the TFs that interact with them (regulators in *trans*).

Non-coding sequences of orthologous genes diverge rapidly during evolution, except for those that are functionally important. This divergence in promoter sequences can be exploited to identify conserved sequences important for the regulation of gene expression, which reduces the need for time-consuming promoter analyses involving random deletions to generate promoter variants. Comparing the sequences of orthologous promoters from several related species increases the evolutionary divergence available and enables reliable detection of conserved non-coding elements whilst still allowing easy alignment of the sequences, an approach that has been called “phylogenetic shadowing” [4]–[6]. Phylogenetic shadowing was shown to be valuable in the identification of known as well as novel conserved motifs that are functionally important in various *A. thaliana* promoters [7]–[12]. The sequences identified by phylogenetic shadowing contain a combination of several *cis*-elements, providing valuable information on the conservation of the core and flanking sequences and pinpointing the different elements to consider when studying the regulation of a given promoter.

TF proteins have a modular structure with a DNA-binding domain (DBD) and a regulatory domain. They are classified into families according to sequence similarities of their DBDs, and some families with plant specific roles are not found in other eukaryotic organisms [13]–[14]. Plants devote a large proportion of their genome capacity to transcriptional control and around 1,500 genes in *A. thaliana* are estimated to encode TFs, representing 6–7% of the genome [13], [15]–[19]. Although many of the characterized TFs have been isolated based on mutant phenotypes, these approaches have limitations because many TFs belong to large families, which often leads to functional redundancy. DNA-protein interactions can be detected in eukaryotic cells by using the yeast one hybrid (Y1H) system. It derives from the original yeast two hybrid (Y2H) method [20] and detection is based on the interaction of a prey TF with a bait DNA-sequence cloned upstream of a reporter gene. When cDNA expression libraries are used as preys, a limitation is that low abundant messengers, such as those derived from many TF encoding genes, tend to be underrepresented. In recent years, the availability of the *A. thaliana* genome sequence and cloning systems based on recombination, have greatly facilitated the generation of several TF open reading frame (ORF) collections that can be used to overcome this limitation [2], [16], [21]–[28].

In this work, we have identified a short promoter fragment from a lipase gene (*LIP1* element) by phylogenomic analyses which was shown to enhance the expression of a minimal promoter-reporter construct *in planta*. Then, we generated an arrayed yeast library of ca. 1,200 *A. thaliana* TF ORFs by extending that generated previously under the REGIA project [2]. The TFs in this library (REGIA + REGULATORS; RR library) are fused to the GAL4 activation domain (GAL4AD) into a Gateway compatible plasmid and were introduced into yeast and individually arrayed in 96-well plates. An episomal plasmid to clone bait sequences has been constructed (pTUY1H) and a mating liquid assay compatible with the arrayed library has been implemented to perform screenings with less than ten hours of labour spread throughout one week. As a proof of principle, we have uncovered novel as well as known DNA-TF interactions by screening the RR library using two promoter fragments selected by phylogenetic shadowing as baits. First, we identified a homeobox TF (HD-ZIP) that specifically binds to a *L1-box* sequence present in the *LIP1* element but not to a mutated version carrying 2 bp changes. This interaction induces the expression of a reporter gene construct *in planta*. Second, we reproduced the previously published interaction of the PHR1 TF, a central regulator of phosphate starvation responses in *A. thaliana*, with a conserved promoter fragment from the *IPS1* gene containing its cognate *cis*-element (P1BS) [12], [29]. Finally, we demonstrated that binding of other members of the HD-ZIP and PHR1 TF families that are present in the RR library reflect their relatedness and functional redundancy in the plant. Thus, our results suggest that phylogenomics coupled to Y1H screenings is a powerful approach to unravel *cis*-*trans* regulation that may also be used to filter genetic redundancy based on DNA-binding specificity of Arabidopsis TFs.

## Results

### Identification of functionally relevant promoter sequences to be used for yeast one hybrid screening

**Figure 1:**
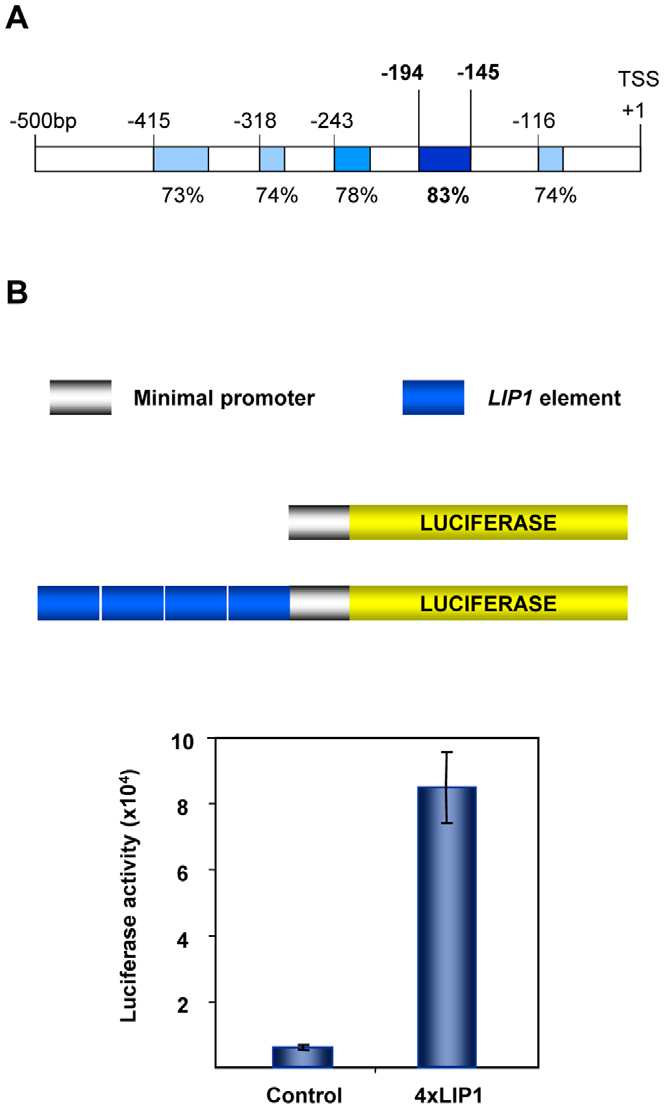
Identification of functionally relevant promoter *cis*-elements in a GDSL-lipase gene from *A. thaliana*. (**A**) The promoter of the *A. thaliana* GDSL-lipase gene (At5g45670) and their orthologous promoters in other *Brassicaceae* species were subjected to *in silico* analysis to identify conserved sequences (shaded boxes). Although over 1 Kb of each promoter was analyzed, significantly conserved sequences were found only along the first 500 bp upstream of the translation start site (TSS). The sequence with the highest degree of conservation (83% identity) is represented as a dark blue box and spans 50 bp (*LIP1* element). (**B**) A binary plasmid (pYRO) containing a minimal promoter fused to the luciferase reporter gene (control) was used to clone four copies of the 50 bp sequence (*4xLIP1*) and both constructs were used to produce *A. thaliana* transgenic plants. Luciferase activity was quantified *in vivo* from transgenic seeds 24 h after imbibition. Average values and standard errors from 10 independent lines for each construct (20 seeds/line) are shown.

We are interested in identifying *cis*-elements involved in the expression control of hydrolase genes putatively involved in the mobilization of seed reserves required for seedling establishment [30]. GDSL-lipases belong to a large protein family of lipolytic enzymes with broad substrate specificity [31] but no information is available regarding their regulatory code. We focused on the promoter of a GDSL-lipase gene (At5g45670; hereafter *LIP1*) that is highly induced upon germination [32]–[34]. *LIP1* orthologous gene promoters from several plant species belonging to the *Brassicaceae* family (*A. thaliana*, *Sisymbrium irio*, *Sinapis arvensis*, *Capsella rubella*, *Descuriainia sophia*, *Brassica oleracea*) were amplified by using a PCR-based approach with degenerated oligonucleotides (see materials and methods). *In silico* analyses of these promoters identified several conserved sequences with over 70% identity and between 25–50 bp in length (Figure 1A and S1). A 50 bp fragment (*LIP1* element) with the highest identity score (83%) was chosen for functional analysis *in planta*. A plasmid containing a minimal promoter upstream of a luciferase reporter gene (*-58F8-pYRO*; control) was used to clone four copies of the *LIP1* element (*4xLIP1-58F8-pYRO*; *4xLIP1*). *A. thaliana* transgenic plants were generated with either the control or the *4xLIP1* construct (Figure 1B) and 10 transgenic lines for each construct (20 seeds each) were used to quantify luciferase expression *in vivo* 24 h after seed imbibition. The *4xLIP1* construct was able to increase luciferase expression 13-fold over the control construct suggesting that the *LIP1* element is being bound by TFs that activate gene expression in germinating seeds (Figure 1B).

### Generation of an arrayed expression library of *A. thaliana* TFs in yeast and construction of the *pTUY1H* plasmid

The strategy to generate a normalized library of *A. thaliana* TF ORFs in yeast is schematically represented in Figure S2. An ORF collection containing 288 TFs (Table S1) was generated (hereafter REGULATORS). This collection does not overlap with that previously generated under the REGIA project [2] but incorporates 71 ORFs found to be mutated or/and truncated in the REGIA collection (Table S1). We have generated a database integrating information from both collections (http://urgv.evry.inra.fr/projects/arabidopsis-TF/) that also incorporates other useful features such as an up-to-date list of TF genes in *A. thaliana* grouped by families and, through FLAGdb++ (http://urgv.evry.inra.fr/projects/FLAGdb/HTML/index.shtml) [35], links to TF transcriptomes from CATdb [36] and GENEVESTIGATOR [37] databases, as well as links to full-length cDNAs, T-DNA insertion mutants and miRNAs.

Nested PCR with appropriate oligonucleotides (Table S2) and a high fidelity polymerase (Pfx; Invitrogen) was used to amplify TF ORFs flanked by *attB* sites that were recombined into a donor plasmid containing *attP* sites (pDONR™221; Invitrogen). Sequencing results (Table S1) confirmed the suitability of our strategy since we found that most ORFs matched their annotated cDNAs or predicted sequences and were free of PCR derived errors. After amplifying 288 ORFs with an average size of 917 bp for 45 cycles, only 21 nucleotide changes, presumably derived from the PCR, were found. Five of these did not produce any amino acid change, six produced a conservative change and ten a non-conservative one (Table S1). We also found five discrepancies with previously annotated ORF sequences likely to represent a bona fide new gene model (Table 1 and S3). Together, both collections (REGIA + REGULATORS; RR library) contain 1,172 unique TF ORFs representing 43 different TF families (Table 2). The TF ORFs were recombined into a yeast compatible plasmid conferring auxotrophy to tryptophan (W) as C-terminal translational fusions to the GAL4AD (pDEST™22; Invitrogen). After verifying the quality of the resulting plasmid collection, clones were individually introduced into yeast (*Saccharomyces cerevisiae* YM4271; A mating type) and the resulting yeast library was stored as glycerol stocks in a 96-well plate format (Figure S2).

**Table 1:** ORFs showing differences with their corresponding sequences present in databases.

		Database		Regulators		
Locus	Family	bp	a.a.	bp	a.a.	Comments
At3g24520	HSF	993	330	990	329	Codon missing
At2g33550	Trihelix	945	314	936	311	Three codons missing
At4g31620	B3	1479	492	1485	494	Two extra codons
At5g49230	ZZ	636	211	621	206	Five codons missing
At5g67580	MYB	900	299	997	190	Intron found. Premature STOP.

**Table 2:** Number of members in the TF families represented in the yeast library.

ABI3/VP1 (14)	AP2/EREBP (124)	ARF (9)	ARR (3)
AUX-IAA (23)	B3 (21)	BES1/BZR (4)	bHLH (87)
Bromodomain (2)	BTB/POZ (2)	bZIP (63)	C2H2 (37)
C3HC4 (44)	CCAAT (24)	CCHC (7)	CCCH (1)
CO-like (31)	Control (29)	DC1 (3)	DOF (33)
EIL (2)	G2-like (34)	GATA (29)	GRAS (28)
HMG (8)	Homeobox (66)	HRT (2)	HSF (18)
MADS (83)	MYB (150)	NAC (77)	PcG (2)
PHD (3)	SBP (12)	SET (6)	TCP (23)
Pseudo-retro (2)	TFII (9)	Trihelix (7)	Unique (2)
WD-40 (6)	WRKY (61)	YABBY (4)	ZZ (6)

The episomal plasmid *pTUY1H* (FR729480) was generated to clone functionally relevant *cis*-elements to use them as baits for Y1H screening with our RR library (Figure S3 and materials and methods).

### Establishment of a protocol for Y1H screenings with the RR library

**Figure 2:**
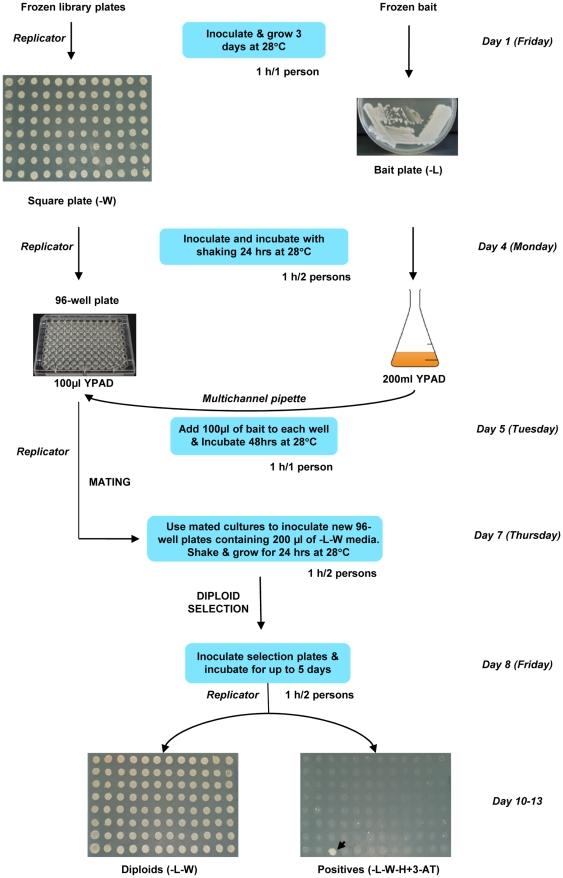
Flowchart for the yeast screening procedure for the arrayed TF library in 96-well format. TF library and bait clones are grown on plates with their corresponding auxotrophic media. These plates were used to inoculate either 96-well plates (TF library; preys) or Erlenmeyer flasks (*cis*-element in *pTUY1H*; bait) containing YPAD and incubated overnight. Bait and preys were then mixed and incubated for 48 h without shaking to allow mating. Mated cells were used to inoculate another set of 96-well plates containing diploid selection media (DOB-L-W). After incubation for 24 h, diploid cells were replicated onto diploid and screening (DOB-L-W-H ±3-AT) plates. Positives were visible after 2 to 5 days of growth. Hours of labour per person are indicated for each step of the protocol.

Several methodological developments were required to perform screenings with an arrayed library in a 96-well format and are outlined in Figure 2. Growing yeast stocks on the corresponding auxotrophic media plates just before using them as inocula to start the screening was crucial to maintain bait and prey plasmids in the yeast cells. Bait clones were grown in Erlenmeyer flasks and the RR library preys in 96-flat bottom well plates in conditions that allowed library preys to grow as fast as bait strains so that, when equal volumes from both cultures were mixed and incubated for 48 h at 28°C, 100% mating was obtained. Mated cultures were used to inoculate a new set of 96-well plates with selection media where only diploid cells could grow. After one day of incubation, diploid enriched cultures were then spotted onto agar plates containing the appropriate media to score for diploids and positive bait-prey interactions, respectively. Although this enrichment step was not essential, it allowed comparable diploid cell densities between wells to be obtained and thereby helped to visually compare the strength of different positive interactions. Moreover, since diploids were maintained under nutritional selection, these plates could be incubated longer than 24 h or stored at 4°C before the spotting step to suit the time schedule of the researcher.

### Identification of a TF binding to the *LIP1* element

**Figure 3:**
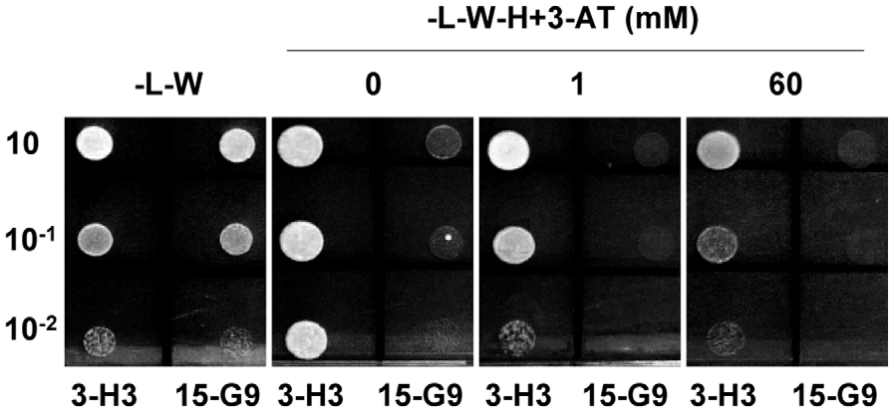
Yeast one hybrid screening with the *LIP1-pTUY1H* construct. Growth of diploid cells at different concentrations of 3-AT from clones showing positive (3-H3) and negative (15-G9) interactions in the screening. Three serial dilutions of diploid cells from saturated cultures were plated.

To screen the RR library, one copy of the *LIP1* element was inserted into the *pTUY1H* plasmid (*LIP1-pTUY1H*) and introduced into *S.cerevisiae* Y187α. Leaky expression of the *HIS3* reporter gene was titrated by using diploid cells obtained after mating the strain containing the *LIP1-pTUY1H* construct (Y187α) with the RR library strain (YM4271) containing a *GFP-pDEST*™*22* construct (*AD-GFP*). Growth of diploid cells was suppressed by using 1 mM 3-AT, a competitive inhibitor of the product of the *HIS3* gene, and therefore this concentration was used in the RR library screen. Only one positive clone was identified in well H3 from library plate 3 (hereafter 3-H3). Cells derived from all wells from plate 3 were able to form diploids and grow at similar densities on diploid plates (Figure 2). However, only diploid cells from 3-H3 were able to grow in media that selected for a positive DNA-protein interaction (screening plates + 1 mM 3-AT; Figure 2). To confirm the interaction, diploid cells from 3-H3 and 15-G9 (randomly selected negative control) were grown in liquid diploid media and similar number of cells were used to inoculate diploid plates and screening plates with increasing amounts of 3-AT. Diploid cells from 3-H3 and 15-G9 (both containing the *LIP1-pTUY1H* construct) produced colonies on diploid plates, but only those containing the *1xLIP1-pTUY1H* and the 3-H3 ORF constructs were able to grow on screening plates even at 60 mM 3-AT, while growth of the negative control was completely blocked at 1 mM 3-AT (Figure 3). According to its position in the library plate, the ORF construct responsible for the activation of the *HIS3* reporter gene contained a class IV homeodomain-leucine zipper gene (*AtML1*; At4g21750). This was also confirmed by sequencing the *ORF-pDEST*™*22* construct present in the diploid cells from 3-H3. The same result observed for diploid cells was obtained when the experiment was repeated using haploid cells of both strains transformed with the appropriate plasmids: 1) The Y187α strain carrying the 1x*LIP1-pTUY1H* construct transformed either with the 3-H3 ORF or the 15-G9 ORF constructs. 2) The YM4271 strain carrying the 3-H3 ORF or the 15-G9 ORF constructs transformed with the 1x*LIP1-pTUY1H* construct. These results ruled out possible effects of the yeast genotype or ploidy on the interaction.

### Binding of AtML1 to a *L1-box* present in the *LIP1* element is abolished by a 2bp mutation and this interaction is relevant in planta

**Figure 4:**
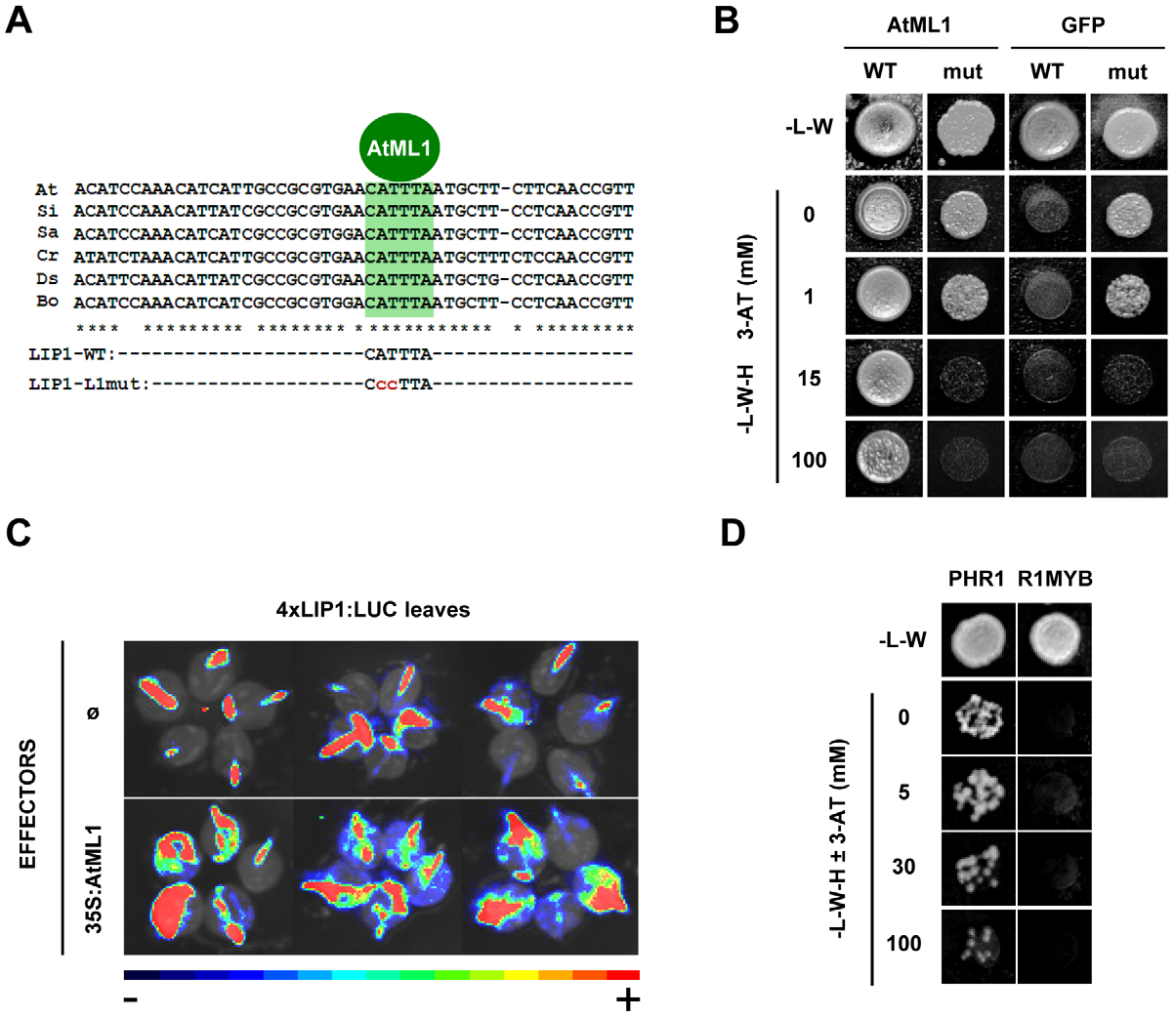
Specificity and *in planta* relevance of the AtML1-*LIP1* element interaction and screening with the *IPS1*-element. (**A**) Alignment of the *LIP1* element (*LIP1wt*) from different *Brassicaceae* species. A conserved *L1-box* sequence putatively bound by AtML1 and other HD-ZIP TFs is shaded. A mutated version of the Arabidopsis *LIP1* element with 2 bp changes in the *L1-box* core sequence was cloned into the *pTUY1H* plasmid (*LIP1-L1mut*). (**B**) AtML1 specifically binds to the *L1-box* sequence of the *LIP1* element. Yeast strains containing either the *LIP1* element (WT) or a 2 bp mutation in the *L1-box* (mut), were mated to strains containing the *AD-AtML1* or *AD-GFP* (negative control) constructs. Diploid cells were grown on auxotrophic media with increasing concentrations of 3-AT. (**C**) The *LIP1* element is an AtML1 target *in planta*. Leaves from transgenic plants carrying the *4xLIP1-58F8-pYRO* construct were bombarded with an empty plasmid (top images) as a control or with a *35S:AtML1* construct (bottom images). Bioluminescence images from three independent experiments are shown. (**D**) A promoter fragment from the *IPS1* gene containing a phosphate starvation responsive element is bound by PHR1 (R1MYB) but not by other R1MYB TF. A strain containing the *IPS1* promoter fragment was mated to strains containing the PHR1 or another family protein (negative control). Diploid cells were grown as in part (B).

The AtML1 protein specifically binds to a motif with a conserved 6 bp core sequence (*L1-box*: 5-TAAATG-3′) and a two base pair mutation in the *L1-box* abolishes both binding of AtML1 *in vitro* and reporter gene expression in transgenic plants [38]. We found that the *LIP1* element contains a *L1-box*, a finding compatible with the positive interaction observed with AtML1 (Figure 4A). To demonstrate that AtML1 binds to the *L1-box* present in the *LIP1* element and in order to test the specificity of the Y1H system, we prepared a mutated version of this element containing two nucleotide changes in the core of the *L1-box* (*LIP1-L1mut*; Figure 4A). The mutated and wild type constructs were introduced into a yeast strain containing the *AtML1-pDEST*™*22* (*AD-AtML1*) or the *AD-GFP* (negative control) constructs and growth of the resulting transformants were scored on plates containing increasing concentrations of 3-AT. When using *AD-GFP*, yeast cells carrying the *LIP1-L1mut* construct required 15mM 3-AT to suppress the basal activity of the reporter gene instead of the 1mM required when they contained the *LIP1-WT* construct (Figure 4B). However, when the *AD-AtML1* construct was used in combination with the *LIP1-WT* construct, growth was observed even at 100 mM 3-AT while growth of cells carrying the *LIP1-L1mut* ceased at 15 mM 3-AT as observed for the control (Figure 4B). These results indicate that AtML1 binds specifically to the *L1-box* sequence present in the *LIP1* element.

To demonstrate that the *LIP1* element is an AtML1 target *in planta*, leaves from transgenic plants carrying the *4xLIP1-58F8-pYRO* construct were bombarded with a *35S:AtML1* construct or with an empty plasmid as a control. Luciferase activity increased markedly when the AtML1 construct was used (Figure 4C). This experiment demonstrates that AtML1 can activate expression from the *LIP1* element in plant cells, consistent with the results from the Y1H experiments.

### PHR1 binds to the *P1BS* sequence present in a promoter fragment from a Pi starvation-responsive gene

To further validate the system, we carried out a screen to reproduce a well characterized DNA-protein interaction reported for the Phosphate Starvation Response 1 protein (PHR1; R1MYB TF) with the *P1BS cis*-element, a motif enriched in promoters of phosphate (Pi) starvation induced genes [12], [29], [39]. For this purpose, we used a conserved 50 bp promoter fragment (*IPS1* element) from a Pi starvation induced gene (*IPS1*) containing the *P1BS* motif [40] that was identified by phylogenomic shadowing [12]. The promoter fragment was cloned into the *pTUY1H* plasmid, introduced into *S.cerevisiae* Y187α cells and the resulting strain mated with the strain containing the *AD-GFP* construct. After titration of the basal expression of the *HIS3* reporter gene, a screening was performed using appropriate plates without 3-AT and one strong positive was identified in well H7 from library plate 5 (hereafter 5-H7). The ORF construct responsible for the activation of the *HIS3* reporter gene was confirmed by sequencing and, as expected, it was found to contain the *PHR1* coding sequence (*R1MYB*; At4g28610). To confirm this interaction, similar numbers of diploid cells from wells 5-H7 (PHR1) and 5-H6 (a *R1MYB* gene not related to *PHR1* used as negative control; At2g40970) were grown on diploid and screening plates with increasing amounts of 3-AT (Figure 4D). Diploid cells from wells 5-H7 and 5-H6 produced colonies on diploid plates but only those containing the *IPS1-pTUY1H* and the 5-H7 ORF constructs were able to grow on screening plates even at 100 mM 3-AT. Growth of the negative control was blocked as soon as the histidine was removed from the media (Figure 4D).

### The *LIP1* and *IPS1* fragments are differentially bound by HD-ZIP and MYB-CC family members, respectively

**Figure 5:**
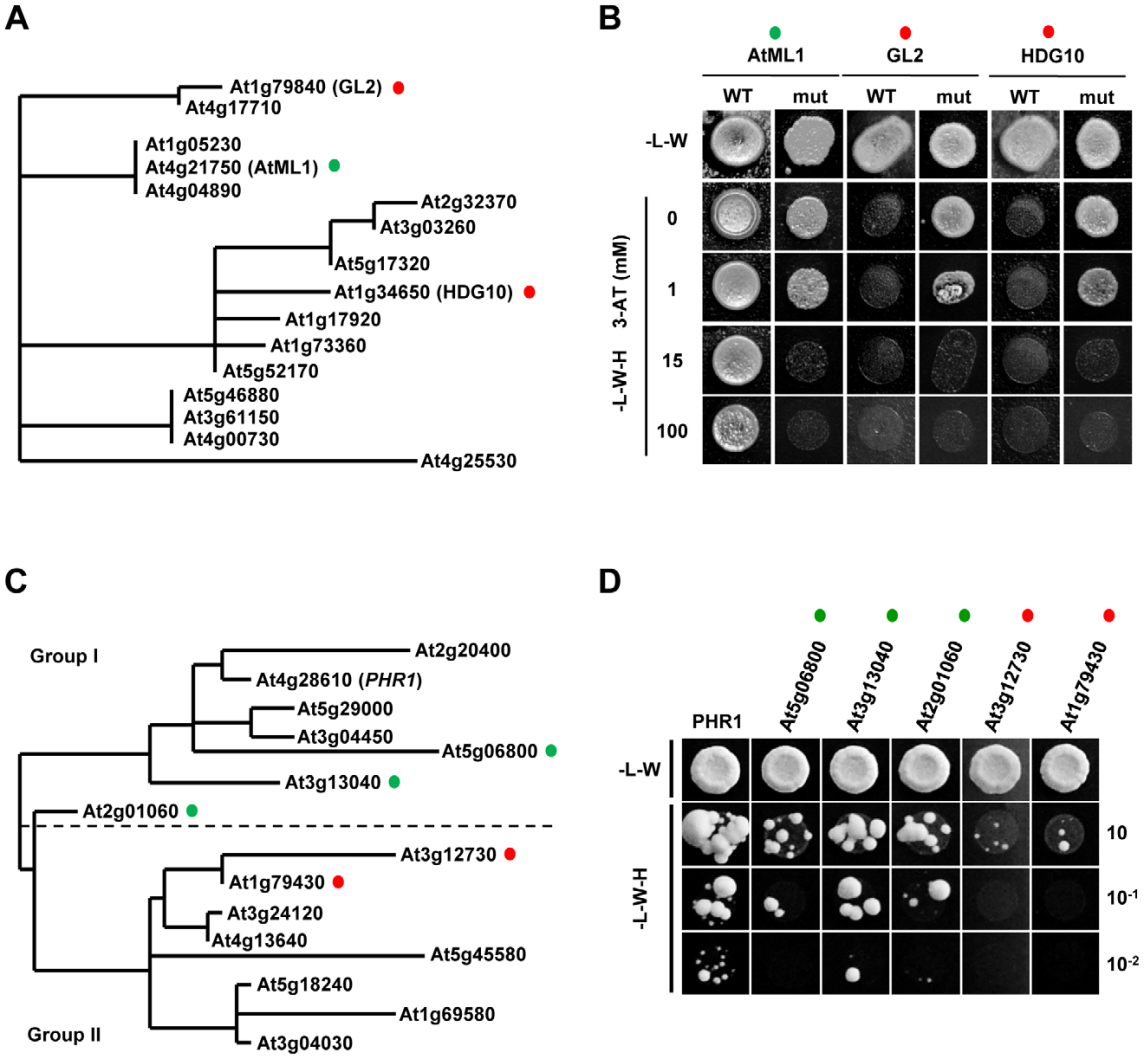
HD-ZIP subfamily IV and MYB-CC proteins show differential binding capabilities. (**A**) Phylogenetic tree of HD-ZIP class IV TF proteins from *A. thaliana* constructed using the Phylogeny.fr platform. TFs used in part (B) are indicated by green and red colored circles. (**B**) Yeast strains containing either the *LIP1* element (WT) or a 2 bp mutation in the *L1-box* (mut), were mated to strains containing the *AD-AtML1*, *AD-GL2* or *AD-HDG10* constructs. Diploid cells were grown on screening plates with increasing concentrations of 3-AT. Only the AtML1 protein is able to activate the reporter gene by binding to the WT element. (**C**) Phylogenetic tree of MYB-CC TF proteins from *A. thaliana* modified from a tree published elsewhere [12]. A dotted line separates Group 1 from Group 2 subfamily members characterized by having the MYB-CC domain at C or N-terminal position, respectively. TFs used in part (D) are indicated by green and red colored circles. (**D**) A yeast strain containing the *IPS1* element was mated to strains containing the *AD-PHR1* or *AD-MYB-CC* TFs. Diploid cells were grown on diploid and screening plates. Only PHR1 and PHR1-like proteins belonging to Group 1 were able to activate the reporter gene .Three serial dilutions of diploid cells were spotted.

AtML1 was the only positive obtained in our screening with the LIP1 element. However, AtML1 belongs to the class IV HD-ZIP TF family that contains members known to bind to the L1-box sequence *in vitro* and *in vivo* [38], [41]-[42]. In our screening, 1mM of 3-AT was used and weaker interactions of the *LIP1* element with other class IV HD-ZIP TFs may have been missed. We used a wider range of 3-AT concentrations to re-examine the ability to bind to the *LIP1* element of GL2 and HDG10, two HD-ZIP proteins present in our library that have different phylogenetic relationships with AtML1 (Figure 5A) as well as different loss-of-function phenotypes and/or expression patterns [41]. As can be seen in Figure 5B, only AtML1 was able to interact with the *LIP1* element even at 100mM 3-AT while GL2 and HDG10 did not activate reporter gene expression even in the absence of 3-AT.

The screening with the *IPS1* element rendered several positives, being PHR1 the strongest one. PHR1 is a R1MYB TF that belongs to the MYB-CC family known to contain 15 members (Figure 5C). According to the functional redundancy observed for members of the PHR1 subfamily [12], other TFs belonging to this subfamily were also able to bind to the *IPS1* element and produced positive interactions in our screening. Re-examination of these interactions showed that only PHR1-like TFs belonging to the MYB-CC group 1 subfamily but not those from group 2 subfamily, were able to bind the P1BS element (Figure 5D).

These results suggest that our system is able to discriminate DNA binding specificities among different members of these TF families according to their functional redundancy in the plant.

## Discussion

In this study we describe an effective approach to decipher DNA-protein interactions underlying transcriptional control in *A. thaliana*. An arrayed library (RR) of ca. 1,200 *A. thaliana* TFs was prepared in yeast and a matrix interaction screening procedure established. We demonstrate that functionally relevant promoter sequences identified by phylogenetic shadowing can be used to screen the RR library to isolate specific DNA binding proteins.

A conserved and functional promoter fragment from a GDSL-lipase gene (*LIP1*) highly induced upon germination was identified by phylogenomic approaches and *in silico* analyses with open access tools (see materials and methods). We used a collection of *Brassicaceae* species with different degrees of phylogenetic closeness with *A. thaliana* to cover a wide range of evolutionary differences and we have applied a new method for isolation of promoter regions based in gene order conservation (synteny). Because of this, we were able to amplify orthologous promoters only in the cases where synteny exists and, high conservation of the ATG and the coding sequence fragments adjacent to the promoter regions isolated, were taken as indicators of orthology [43]–[44]. In case that a wrong orthology had been assigned, this would not invalidate the conclusion on the likely relevance of the conserved boxes identified. Rather this would have potentially resulted in the non-identification of some relevant boxes, i.e., conserved among orthologous genes, but not conserved among closely related genes. Although there are some examples of exceptions in which considerable shuffling and alteration in number of binding sites in enhancers may occur among related species [7], [45]–[49], it is clear that conserved motifs are more likely to be functionally relevant [7]–[12]. With the recent developments in DNA sequencing technology, an increasing number of plant genomes are being sequenced and annotated, thus avoiding the need for experimental promoter isolation and speeding up the discovery of conserved *cis*-regulatory elements.

During the generation of the REGULATORS collection, ca. 2% of the clones showed differences with predicted transcripts or annotated cDNA clones available in TAIR9 (http://www.arabidopsis.org). This was comparable to the discrepancies observed during a similar project conducted by Gong *et al* (2004) and indicates that experimental data for transcripts may still contribute to improve genome annotation. The REGULATORS collection, together with that generated under the REGIA project, used the Gateway-recombination method and contains ca. 1,200 TFs. It contains 469, 319 or 258 new ORFs when compared to the collections generated by Gong *et al* (2004), Mitsuda *et al* (2010) and Ou *et al* (2011), respectively, indicating that they are additive resources.

Y1H and Y2H systems can be used for high throughput studies of DNA-protein and protein-protein interactions. Yeast cells can be used as convenient eukaryotic test systems that require little specific optimization for each interaction compared to other approaches, and are more likely to provide an appropriate environment for interactions that depend on post-transcriptional modifications. Using arrayed TF libraries instead of pooled TF collections, reduce labour time since this eliminates the effort required to characterize several positives produced by the same clone. For instance, Mitsuda *et al* (2010) detected 72 positive interactions with a promoter fragment, being the same TF responsible for 39 of them. In our system, mating is carried out in liquid media so that diploid and screening plates are inoculated with similar numbers of cells and grown and scored in parallel, allowing eventual non-mating clones to be flagged as not screened. Diploid colony size can be taken into account to compare and normalize the strength of positive interactions. Moreover, the 96-well plates containing the diploid cells can be stored at 4°C and re-spotted at any time on different types of screening plates, for instance containing hormones or other chemicals, to re-evaluate positive and negative interactions from the initial screening. The simplicity of the procedure, offers the possibility to easily perform these screens with reduced labour and time. Also, more complex matrix interaction schemes involving several baits can be performed [27], [50]. As an added value, this library constitutes a convenient tool for the plant community since it could also be used for Y2H and Y3H approaches. In fact, the RR library has been successfully used in two hybrid screenings, by using a *pDEST*™*32-ORF* clone in the *S.cerevisiae* PJ694α as bait, to reproduce previously published interactions [51]-[52].

We have shown that a single copy of a discrete promoter fragment can be used with an episomal plasmid in Y1H screenings without compromising specificity. It has been common to Y1H experiments to use large promoter fragments or generate tandemly repeated promoter sequences that need to be integrated into the yeast genome. Using large promoter fragments requires performing additional promoter deletions/mutations and experiments to pinpoint the exact sequence bound by the TF identified. Moreover, *S.cerevisiae* genome is more compact than that of *A. thaliana* and it is known that for UAS located over 300bp upstream of a reporter gene, transcription initiates proximally to the UAS and competes with that derived from the reporter gene located downstream [53]. For instance, this could explain why Brady *et al* (2011) carried out Y1H matrix assays between 167 TFs and 65 promoters (3 kb) mainly expressed in the stele and they only detected positive interactions for 16 promoters. Also, Mitsuda *et al* (2010) used 500 bp promoter fragments for Y1H screenings with a pooled TF library and found that only one out of 2 positive interactions seemed to be biologically relevant when tested *in planta*. Their results suggest that such promoter fragments may be missing information contained in upstream parts of the promoters used and adding noise to the system by increasing the probability of having yeast TF derived positives.

Compared with the use of core *cis*-elements (typically 6–8 bp length), small promoter fragments such as those identified by phylogenomic shadowing, may allow discrimination between DNA binding specificities among different members of a TF family and the identification of several TFs binding to different target sequences, while focusing on a small part of the promoter likely to be involved in its regulation. We have uncovered a novel interaction between a lipase promoter and AtML1, a class IV HD-ZIP protein. AtML1 binds to a L1-box, a motif that is also bound *in vitro* by class IV HD-ZIP proteins [41]. However, the L1-box sequence present in the *LIP1* element exclusively interacted with AtML1 but not with two other class IV HD-ZIP proteins expected to have different functions in the plant (Figure 5B). Also, several TFs belonging to the PHR1 subfamily were able to bind to the *IPS1* element (Figure 5D) according to their functional redundancy and phylogenetic relatedness [12].

Our results suggest that approaches such as the one presented here, which includes a phylogenetic shadowing-based motif identification step, may be more restrictive and specific in detecting relevant DNA-protein interactions. Identifying target DNA binding motifs and their partners is crucial to understand their function and the approach reported here will facilitate future studies on transcriptional regulation of gene expression.

## Materials and Methods

### Cloning orthologous *LIP1* gene promoters from several *Brassicaceae* species and phylogenomic analyses

For the phylogenomic analysis of the *LIP1* gene (At5g45670), a collection of 18 *Brassicaceae* species, that represents a wide evolutionary range inside this family, was used (Figure S4). Using this collection for promoter amplification of orthologous *LIP1* genes, we first designed 32–33 mers oligonucleotide mixtures to be used as primers (LIP1forward and LIP1reverse; Table S2). These oligonucleotides mixtures were derived from the conserved protein coding regions of the *LIP1* gene and the upstream gene flanking the *LIP1* promoter in *A. thaliana* (At5g45660). For each primer, the oligonucleotide mixture reflected all sequence variants encoding these conserved regions as found in *A. thaliana, Brassica oleracea*, *Brassica rapa* and *Brassica napus*. In addition, the 12 nts at the 3′-end of the primer mixture included all possible combinations, according to the genetic code, encoding the corresponding 4 amino acids of the conserved protein sequences. For all the promoter amplifications the Expand long template PCR system (Roche) was used. Cycling conditions were 5 min at 95°C, 10 cycles of 10 s at 95°C, 5 min 30 s at 68°C followed by 20 cycles of 15 s at 95°C, 5 min 30 s + 10 s/cycle at 68°C. Out of the *Brassicaceae* species in which clear PCR amplification products was observed, five of them displaying different degrees of phylogenetic closeness with *A. thaliana* were selected for sequencing. PCR products were separated by agarose electrophoresis, purified (QIA quick gel extraction kit, Qiagen) and cloned using the pCRII-TOPO TA Cloning Kit (Invitrogen).

Alignment of promoter sequences was performed by using DiAlign (http://www.genomatix.de/cgi-bin/dialign/dialign.pl) [54], a program with a high resolving power in the detection of conserved blocks. High conservation of the ATG and the coding sequence fragments adjacent to the promoter regions isolated were taken as indicators of orthology [43]–[44].

The phylogenetic tree of the HD-ZIP class IV proteins was performed on the Phylogeny.fr platform (www.phylogeny.fr) [55] as previously described [12]. The default substitution model was selected assuming an estimated proportion of invariant sites (of 0.076) and 4 gamma-distributed rate categories to account for rate heterogeneity across sites. The gamma shape parameter was estimated directly from the data (gamma  = 0.321). Nodes with bootstrap value <30 were collapsed. Only the conserved DNA binding domain was used for alignment and tree construction.

### Generation of constructs for plant transformation and *in vivo* imaging of transgenic plants

A 969 bp promoter fragment from the lipase gene was amplified by PCR from *A. thaliana* (Columbia ecotype) genomic DNA using the LO843 and LO848 primers (Table S2) and cloned into the pGEM®-T Easy vector (Promega). This construct was used as a template to amplify a 50 bp promoter sequence (*LIP1 element*; -194-145 in Figure 1A) using the primers LO846 and LO847 that contained *Bgl2* and *BamH1*/*Hind3* restriction sites, respectively (Table S2). To introduce four tandemly repeated *LIP1 elements* into plants, we used a promoterless binary plasmid containing a luciferase (*LUC+*) encoding sequence (*pYRO*) [56]. First, we introduced a *GSTF8* gene minimal promoter sequence [57] in front of the *LUC+* reporter gene (*-58F8-pYRO*). To do this, we amplified the *GSTF8* minimal promoter with primers -58F8-F (*Bgl2*, *BamH1* and *Hind3*) and -58F8-R (*Sal1*) containing engineered restriction sites. The fragment was digested with *Bgl2* and *Sal1* (filled in after digestion) and cloned into *pYRO* digested with *BamH1* and *Hind3* (filled in after digestion). Then, the engineered *Bgl2* and *Hind3* sites of the *LIP1 element* were used to clone it into the *BamH1* and *Hind3* sites of the *-58F8-pYRO* plasmid upstream of the minimal promoter and the *LUC+* gene (*1xLIP1-58F8-pYRO*). This process was repeated three more times to generate a *4xLIP1-58F8-pYRO* plasmid. The *-58F8-pYRO* and *4xLIP1-58F8-pYRO* constructs were introduced into *A. thaliana* (Col-0 ecotype) using the floral-dip method and the *Agrobacterium tumefaciens* strain GV3101 [58]-[59]. Seeds from these transgenic plants were sown on MS agar plates containing 50 µM of luciferin and stratified for 2 days (4°C in the dark). Luciferase activity was recorded *in vivo* after 24 h of imbibition at a constant temperature of 22°C and a photoperiod of 16 h light/8 h dark. *In vivo* imaging and quantification was performed using a CCD camera (Hamamatsu) and the provided software.

To generate the 35S:AtML1 construct, the AtML1 ORF was transferred from the corresponding yeast library clone (*AD-AtML1*) to the pDONR™221 by recombination (BP clonase; Invitrogen). The AtML1 ORF was then recombined (LR clonase; Invitrogen) into the pEarleygate201 plasmid [60] and the resulting construct checked by sequencing. Transient expression analyses were performed according to [61], except that bombarded leaves were sprayed with a 5mM luciferin solution 15–30 min before imaging.

### Generation of the *pTUY1H* plasmid and cloning of *LIP1* and *IPS1* elements

To generate pTUY1H, a fragment encompassing the *ARS6/Cen4* centromeric region and the *LEU* (L) auxotrophy marker was amplified from the pDEST™32 vector (Invitrogen) using the oligonucleotides yeast-fw and yeast-rv (Table S2). The fragment was subcloned into pPCR2.1-TOPO (Invitrogen) and a *BamHI*-*EcoRV* fragment that contained the full cassette was obtained by partial digest. The fragment was ligated into the *pHISi-1* vector (Clontech) after BamHI and PvuII digestion.

We used the *1xLIP1-58F8-pYRO* construct as a template to amplify the *LIP1* element by PCR using the primers LO1203 and LO1204 that contained *Xma1* and *Xba1* restriction sites, respectively (Table S2). Then, the engineered restriction sites of the *LIP1* element were used to clone it into the *Xma1* and *Xba1* sites of the *pTUY1H* plasmid upstream of the *HIS3* reporter gene. The same restriction sites of the *pTUY1H* plasmid were used to clone a 50 bp fragment from the *IPS1* gene that contained a *P1BS* sequence. This fragment was produced by annealing complementary single-stranded oligonucleotides (IPS1Y1H5 and IPS1Y1H3; Table S2) that generated *XmaI* and *XbaI* 5′- cohesive ends.

### Generation of a TF ORF collection in a Gateway compatible entry vector

288 TF ORFs from *A. thaliana* were amplified by 2-step nested PCR and cloned into a Gateway compatible plasmid (pDONR™221). In the first PCR, ORF specific primers containing half of the *att*B sequences at the 5′-ends (Table S2) were mixed with Pfx polymerase buffer (2x), 1 mM MgSO_4_, 0.2 mM of each dNTP, 0.2 µM of each primer, 0.2 units of Pfx polymerase (Invitrogen) and template in a 10 µl total volume. Three different types of templates were used depending on the ORF (Table S1): 10 ng of SSP clones (Salk), 20 ng of a cDNA mix or 70 ng of genomic DNA. Cycling conditions were 5 min at 95°C, 10 cycles of 15 s at 95°C, 30 s at 55°C and 60 s at 68°C. The time of extension varied depending on the expected ORF size (60 s / Kb). The first PCR reactions were used as templates for a second PCR (50 µl total volume) with oligonucleotide adapters containing the full *att*B sequences (Table S2). PCR and cycling conditions were identical as those used for the first PCR, except for the concentration of the primers (0.8 µM), the amount of the Pfx polymerase (0.8 units) and the number of cycles (35). PCR products were subjected to electrophoresis, purified (PEG/MgCl_2_ solution, Invitrogen or QIA quick gel extraction kit, Qiagen) and cloned into the pDONR™221 plasmid (Invitrogen) using the BP clonase mix (Invitrogen). Integrity and quality of every ORF was checked by sequencing using M13 forward and reverse primers (Table S2).

### Generation of a yeast normalized library of A. thaliana TFs

Entry clones (pDONR™221-TF ORF) were recombined to the pDEST™22 plasmid using the Gateway LR enzyme (Invitrogen) to generate GAL4AD-ORF fusions. Reactions were prepared by mixing 25 ng Entry-clone, 75 ng pDEST™22, 0.5 µl LR buffer and 0.4 µl LR clonase and adjusting the volume up to 2.5 µl with TE. After overnight incubation in thin-walled 96-well tubes, 10 µl library efficient chemically competent cells (E.coli DH5α; Invitrogen) were added and transformed with the heat-shock method (15 s, 42°C). Positive colonies were selected by colony PCR according to size using pD22f and pD22r primers (Table S2) and plasmids were purified using a high-throughput 96-well robotic system. Plasmids were further verified by analytical digest using *BsRGI* restriction endonuclease and individually transformed into the yeast strain YM4271. Competent yeast cells were prepared by growing a yeast suspension (250 ml) started from a fresh overnight culture (5 ml) in YPD-medium (30°C, 220 rpm, 4–5 h). All the following steps were performed at room temperature (RT) unless stated otherwise. Cells were harvested in 50 ml aliquots (20 min, 4000 rpm) by centrifugation, washed once in sterile H_2_O (25 ml per falcon tube, 20 min, 4000 rpm) and then resuspended in 1 ml of 100mM lithium acetate (LiAc) pH 7.5. Yeast cells were transferred to 1.5 ml eppendorf tubes, pelleted by centrifugation (30 s, 11000 g) and resuspended in 1ml of fresh LiAc. Yeast suspensions from different aliquots were then pooled and distributed into flat bottom 96-well plates (100 µl/well). The plates were centrifuged (5 min, 4000 rpm) and the supernatant from each well discarded. 340 µl of a LiAc/PEG4000 solution (240 µl of 50% PEG4000, 36 µl of 1M LiAc pH 7.5, 25 µl of 2mg/ml ssDNA and 50 µl H_2_O) and 2 µl of each plasmid (70–150 ng/µl) were added to each well and the yeast pellet resuspended by repeated pipetting. The suspensions were first incubated for 25 min without shaking and then heat shock treated for 25 min at 42°C. Cells were pelleted by centrifugation (5 min, 4000 rpm, 42°C), resupended in 100 µl of 1M sorbitol and plated on 6 cm selection plates (minimal medium minus tryptophan, DOB-W; MP Biomedicals). One positive colony per transformation was used to inoculate deep 96-well plates containing 1.5 ml of DOB-W, and grown for 48 h at 30°C and 250 rpm. Frozen glycerol stocks were prepared by mixing 100 µl of yeast cultures with 100 µl of 50% glycerol prior freezing at −80°C. To further asses quality of the RR library and efficiency of the recombination, 64 randomly picked clones were re-sequenced (Table S4). Detailed information of the collection can be downloaded from the REGULATORS website (http://urgv.evry.inra.fr/projects/arabidopsis-TF/). The RR yeast library (15 x 96-well plates) will be made available to the research community through the NASC repository.

### Protocol for yeast screenings

Library and bait clones were grown for three days on DOB-W and DOB-L (MP Biomedicals) plates from their corresponding frozen stocks. A 1 L Erlenmeyer flask containing 200 ml of YPAD medium was inoculated with a clump of bait cells (5–10 colonies) and incubated overnight at 28°C with shaking (200 rpm). In parallel, 100 µl of YPAD was aliquoted into 96-flat bottom well plates (Corning) by using a multichannel pipette and a replicator was used to inoculate them with their corresponding library colonies. After overnight incubation with vigorous shaking (500 rpm) at 28°C (HiGro shaker; Genemachines), 100 µl of the bait culture was added to each well of the 96-well plates with a multichannel pipette and mating was allowed 48 h by incubating at 28°C without shaking. Settled cells were resuspended by hitting the bottom of the wells with the pins of the replicator and used to inoculate another set of 96-flat bottom well plates containing 200 µl of diploid selection media (DOB-L-W). The replicator was able to transfer about 5 µl of liquid in each pin and each well was inoculated twice (1/20 dilution), always sterilizing by flaming with absolute alcohol between plates. After one day of growth at 28°C and vigorous shaking, diploid cells were resuspended with the replicator and spotted onto diploid selection and screening (DOB-L-W-Histidine ±3-Amino-1,2,4-triazole; 3-AT, Sigma) plates. Positive colonies were visible after 2 to 5 days of growth at 28°C.

## Supporting Information

Figure S1 **Alignment of** ***LIP1*** **proximal promoter regions from different** ***Brassicaceae*** **species.** Sequences from *Arabidopsis thaliana, Sisymbrium irio* (HQ322377), *Sinapis arvensis* (HQ322378), *Capsella rubella* (HQ322379), *Descurainia Sophia* (HQ322380) and *Brassica oleracea* (HQ322381) were aligned using DiAlign (http://www.genomatix.de/cgi-bin/dialign/dialign.pl) [54]. A conserved region among *LIP1* orthologs is shadowed in grey and the *L1-box* included in this region is highlighted in yellow. GenBank accession numbers are shown in parenthesis.
(TIF)

Figure S2 **Construction of an Arabidopsis TF collection and generation of an arrayed yeast library.** A collection of TF ORFs (REGULATORS) was generated by PCR using a high fidelity polymerase and nested primers, and recombined into the pDONR™221 plasmid. The REGULATORS and REGIA collections were recombined into the pDEST™22 plasmid and introduced into yeast. Clones were arrayed separately in 96-well plates and maintained as glycerol stocks at −80°C.
(TIF)

Figure S3 **Map of the pTUY1H plasmid.** Modifications made on the pHISi-1 backbone are shown: ARS6/Cen4 centromeric region, LEU auxotrophy marker and the multicloning site.
(TIF)

Figure S4 ***Brassicaceae*** **species used for the amplification of** ***LIP1*** **orthologous gene promoters.** (A) Table containing the tribes and species of the *Brassicaceae* collection. Species marked with the letter A inside a red circle were used for the phylogenetic analysis of *LIP1*. (B) Synoptic diagram of phylogenetic relationships of *Cleomaceae* and the various tribes of the *Brassicaceae*, adapted from [62]. The tribes in red represent the ones used in the promoter regions isolation of different orthologous of the *LIP1* gene.
(TIF)

Table S1 **List of TF ORFs in the REGULATORS collection.**
(XLS)

Table S2 **List of oligonucleotides used in the paper.**
(XLS)

Table S3 **Full coding sequences corresponding to ORFs that differ with their annotated sequences in databases.**
(DOC)

Table S4 **Sequencing results of randomly picked RR library clones.**
(XLS)
