# Supplementary figures and images for: Speeding Cis-Trans Regulation Discovery by Phylogenomic Analyses Coupled with Screenings of an Arrayed Library of Arabidopsis Transcription Factors
Source: PLoS One. 2011 Jun 27;6(6):e21524. doi: 10.1371/journal.pone.0021524 (PMC3124521; doi:10.1371/journal.pone.0021524)

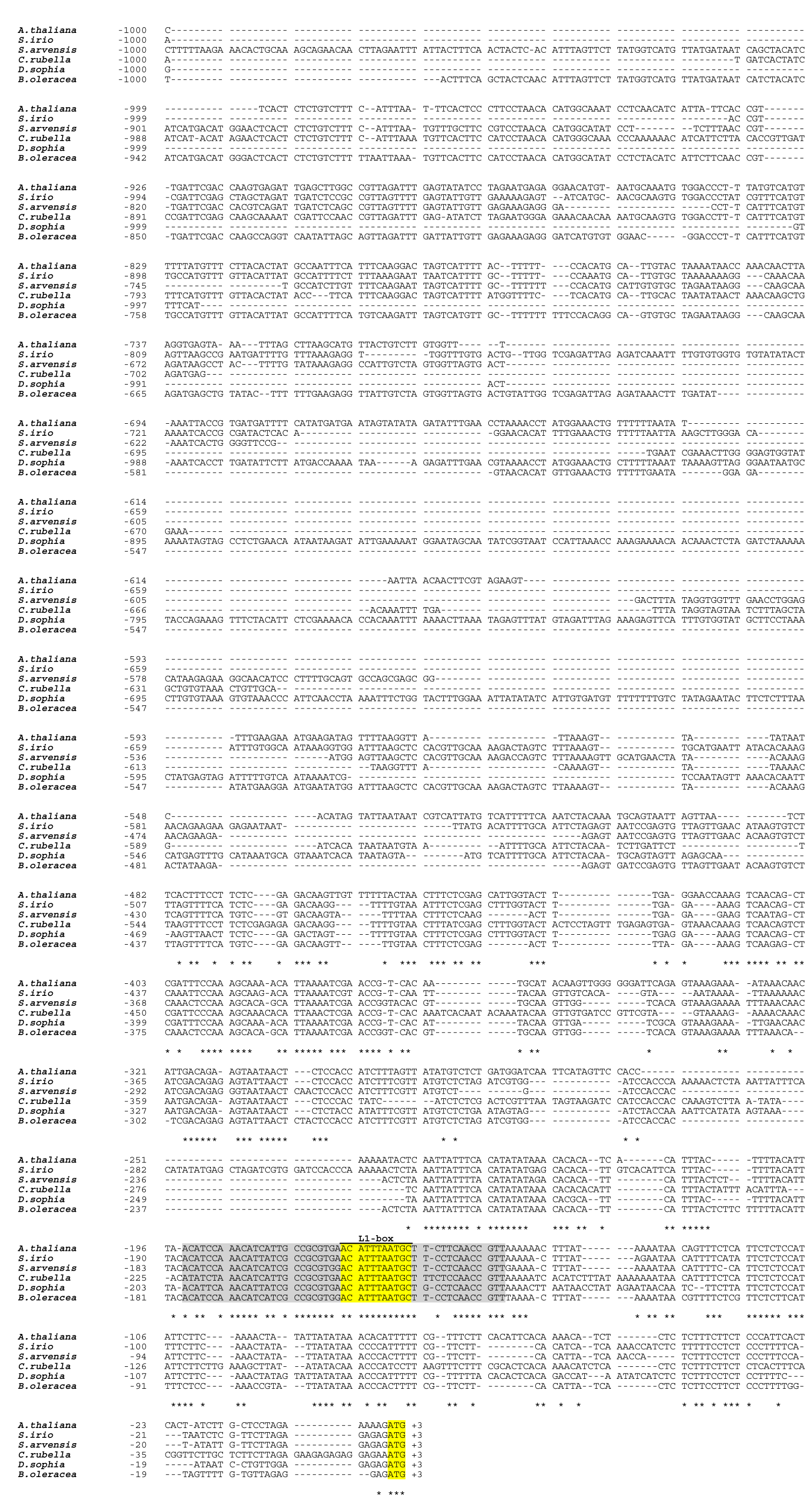

Supplement: Figure S1 — Alignment of LIP1 proximal promoter regions from different Brassicaceae species. Sequences from Arabidopsis thaliana, Sisymbrium irio (HQ322377), Sinapis arvensis (HQ322378), Capsella rubella (HQ322379), Descurainia Sophia (HQ322380) and Brassica oleracea (HQ322381) were aligned using DiAlign (http://www.genomatix.de/cgi-bin/dialign/dialign.pl) [54]. A conserved region among LIP1 orthologs is shadowed in grey and the L1-box included in this region is highlighted in yellow. GenBank accession numbers are shown in parenthesis. (TIF) [file pone.0021524.s001.tif]

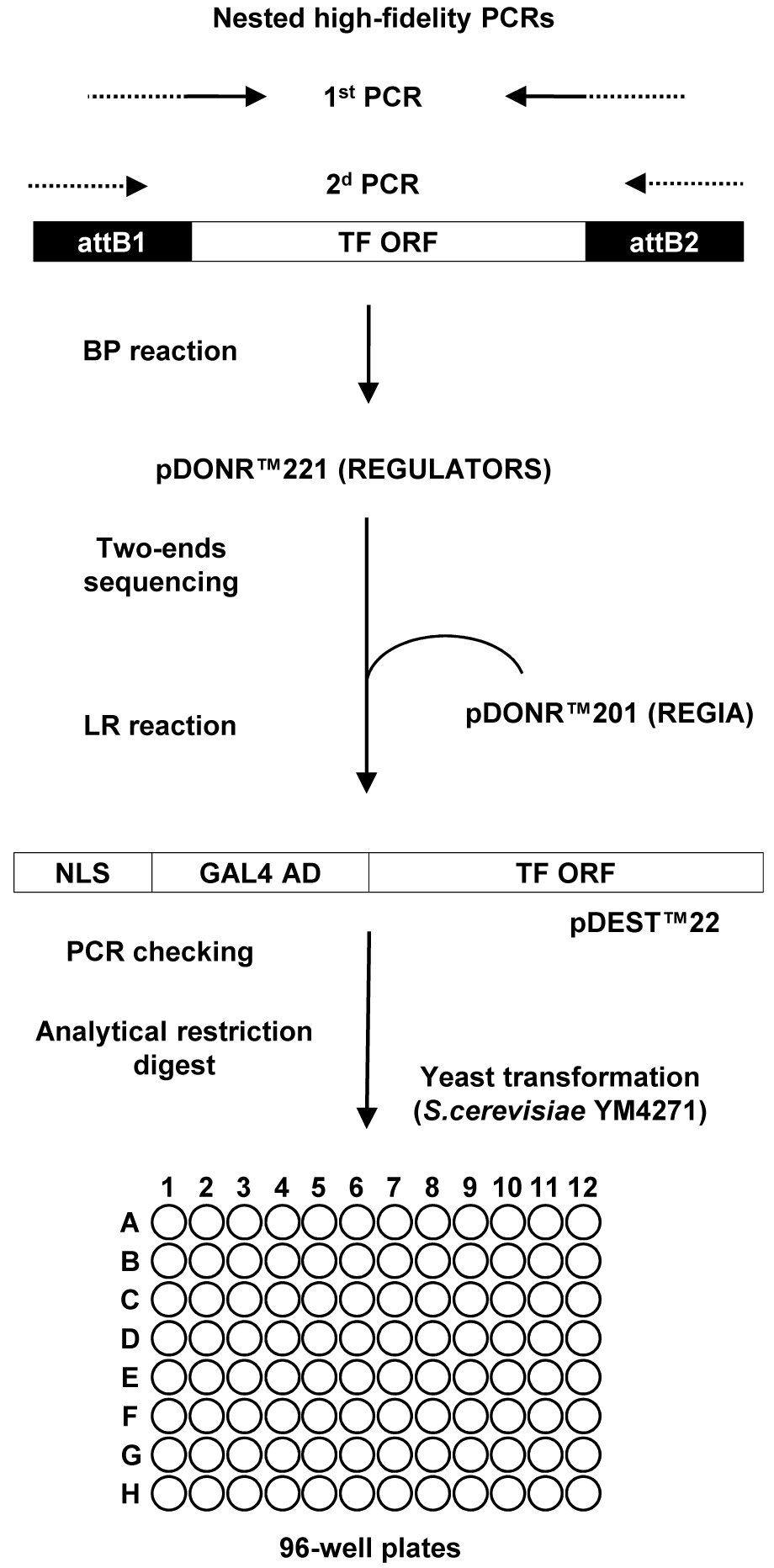

Supplement: Figure S2 — Construction of an Arabidopsis TF collection and generation of an arrayed yeast library. A collection of TF ORFs (REGULATORS) was generated by PCR using a high fidelity polymerase and nested primers, and recombined into the pDONR™221 plasmid. The REGULATORS and REGIA collections were recombined into the pDEST™22 plasmid and introduced into yeast. Clones were arrayed separately in 96-well plates and maintained as glycerol stocks at −80°C. (TIF) [file pone.0021524.s002.tif]

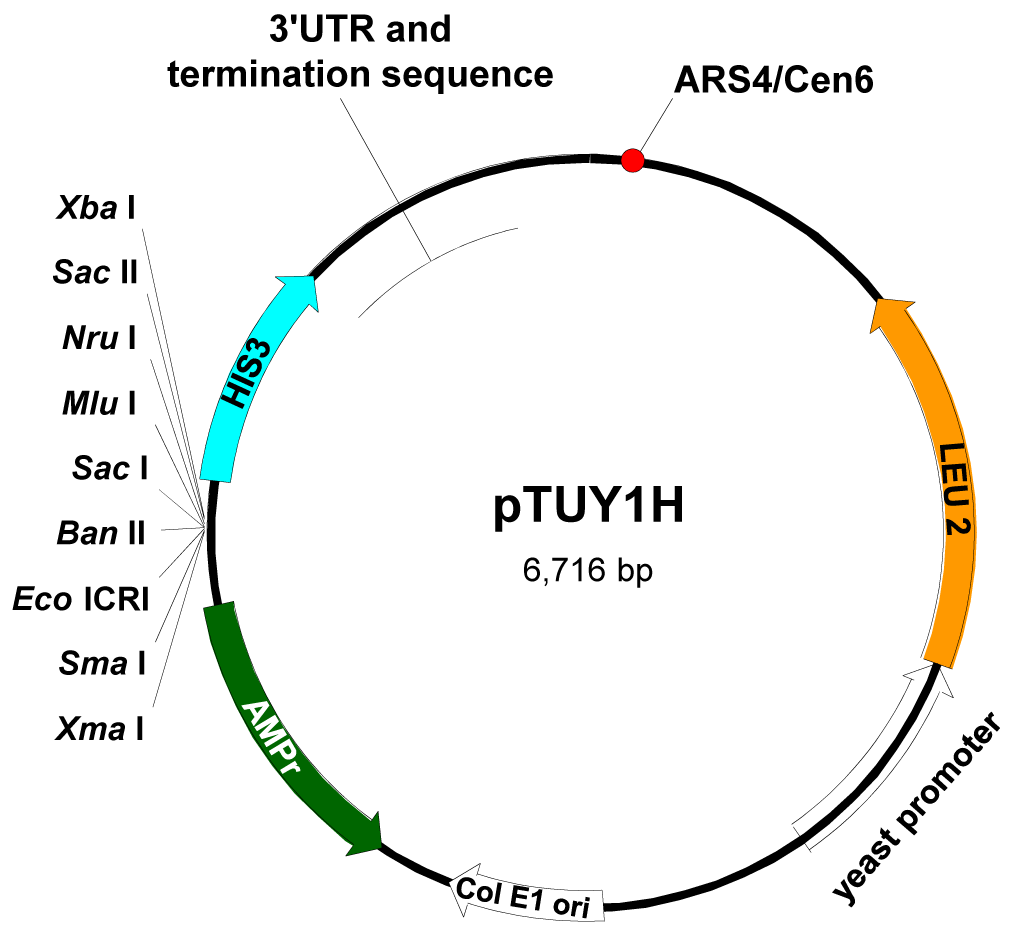

Supplement: Figure S3 — Map of the pTUY1H plasmid. Modifications made on the pHISi-1 backbone are shown: ARS6/Cen4 centromeric region, LEU auxotrophy marker and the multicloning site. (TIF) [file pone.0021524.s003.tif]

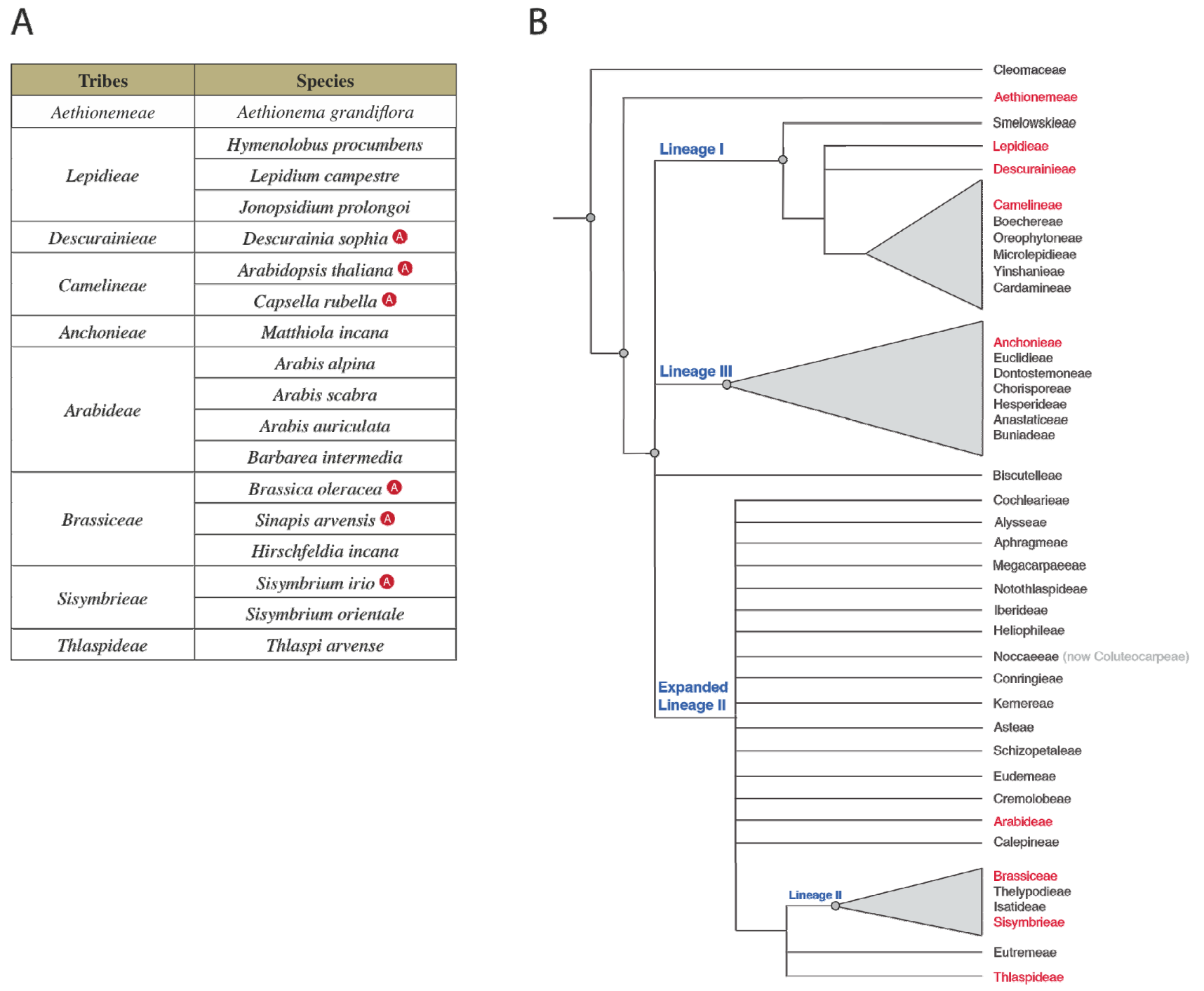

Supplement: Figure S4 — Brassicaceae species used for the amplification of LIP1 orthologous gene promoters. (A) Table containing the tribes and species of the Brassicaceae collection. Species marked with the letter A inside a red circle were used for the phylogenetic analysis of LIP1. (B) Synoptic diagram of phylogenetic relationships of Cleomaceae and the various tribes of the Brassicaceae, adapted from [62]. The tribes in red represent the ones used in the promoter regions isolation of different orthologous of the LIP1 gene. (TIF) [file pone.0021524.s004.tif]
