# Supplementary material for: Speeding Cis-Trans Regulation Discovery by Phylogenomic Analyses Coupled with Screenings of an Arrayed Library of Arabidopsis Transcription Factors
Source: PLoS One. 2011 Jun 27;6(6):e21524. doi: 10.1371/journal.pone.0021524 (PMC3124521; doi:10.1371/journal.pone.0021524)
Supplement: Table S3 — Full coding sequences corresponding to ORFs that differ with their annotated sequences in databases. (DOC) [file pone.0021524.s007.doc]

**Table S3.** **Full coding sequences corresponding to ORFs that differ with their annotated sequences in databases.**

**Green: Accession numbers for ORF sequences corresponding to new gene models are shown in parenthesis.**

**START and STOP codons in bold.**

**Red: Missing in ORF but present in the predicted sequence from databases.**

**Blue: Present in the amplified sequence but absent in sequence from databases.**

At3g24520 (HSF) 993bp Template: cDNA. Clone: U14335. The protein will be 1 aminoacid shorter. (**HQ322382**)

**ATG**GAGGACGACAATAGTAACAACAACAACAACAACAACGTGATCGCACCTTTCATCGTGAAAACATATCAGATGGTCAATGATCCTTCCACCGATTGGCTCATCACTTGGGGACCTGCTCACAACAGTTTCATAGTCGTTGATCCACTCGACTTCTCGCAACGAATCTTACCTGCTTATTTCAAACACAACAATTTCAGCAGTTTTGTTCGTCAACTCAACACCTATGGGTTTAGGAAAGTGGATCCGGACCGGTGGGAGTTTGCGAACGAGCATTTTCTAAGGGGACAGAAGCATTTGTTGAATAACATAGCGCGTAGGAAACACGCGCGGGGGATGTACGGTCAAGATTTGGAGGACGGTGAGATTGTGAGGGAGATCGAACGGTTAAAAGAAGAGCAGAGGGAGCTAGAGGCAGAGATTCAGAGGATGAACCGGAGGATCGAAGCGACGGAGAAGAGACCGGAGCAGATGATGGCGTTTCTTTACAAAGTCGTTGAAGATCCTGATCTTCTTCCAAGAATGATGCTCGAGAAAGAACGGACAAAGCAACAACAACAAGTTTCCGACAAGAAGAAGCGTCGTGTCACGATGTCGACGGTGAAGTCAGAGGAAGAAGAAGTGGAAGAAGATGAAGGGAGGGTCTTTAGGGTTATGTCATCATCAACACCGTCGCCGTCATCAACGGAGAATCTTTACCGGAATCATTCACCGGATGGGTGGATTGTTCCTATGACGCAAGGACAGTTTGGTAGTTACGAGACGGGTTTGGTGGCGAAGTCGATGCTCTCGAATTCAACGTCGTCGACGTCATCATCTTTGACGTCGACGTTTTCTTTGCCGGAGAGTGTTAACGGAGGAGGAGGAGGA**GGA**TGTGGGAGTATTCAGGGAGAAAGAAGGTATAAAGAAACGGCGACGTTTGGAGGAGTGGTAGAGTCAAATCCACCAACAACACCGCCTTATCCATTTTCTCTGTTTCGAGGTGGCTTT**TAG**

At2g33550 (Trihelix) 945bp Template: cDNA. Clone: U11592. The protein will be 3 aminoacids shorter. (**HQ322383**)

**ATG**GCTCTGGAACAGTTAGGATTAGGAGTGAGCGCCGTTGACGGTGGAGAGAACAGTAGCGCGCCGTCAAATGACGGTGGAGATGACGGCGTTAAGACGGCGAGACTTCCTCGTTGGACGAGACAAGAGATTCTGGTTCTGATTCAAGGGAAGAGAGTGGCGGAGAACAGAGTCCGGCGAGGGAGAGCGGCGGGTATGGCTCTCGGGTCGGGTCAAATGGAGCCTAAATGGGCTTCTGTTTCGTCTTACTGTAAACGTCACGGTGTAAATCGTGGGCCGGTTCAGTGCCGGAAAAGATGGAGCAATCTCGCCGGAGATTATAAGAAGATTAAAGAATGGGAGTCTCAGATTAAGGAAGAGACTGAGTCCTATTGGGTTATGAGGAATGATGTTCGTAGAGAGAAGAAGCTTCCTGGTTTTTTCGATAAGGAGGTTTATGATATTGTTGACGGTGGTGTGATTCCTCCGGCGGTTCCGGTTCTTTCGCTTGGATTGGCTCCGGCGTCAGACGAGGGATTGTTGTCTGATTTAGATCGGAGAGAAAGTCCTGAGAAGTTGAATTCTACTCCGGTGGCTAAATCAGTTACTGA**TGTTATAGA**CAAAGAGAAGCAAGAAGCTTGTGTAGCAGATCAAGGTAGAGTGAAAGAGAAACAGCCAGAAGCAGCAAACGTGGAAGGTGGATCGACATCACAAGAAGAGAGGAAGCGTAAACGGACATCTTTTGGTGAAAAGGAAGAGGAAGAAGAAGAAGGAGAAACAAAGAAGATGCAGAATCAGTTGATAGAGATACTAGAAAGAAACGGGCAGTTGTTGGCGGCACAGCTTGAGGTTCAGAATTTAAACTTAAAACTAGACAGAGAGCAAAGAAAAGATCACGGTGATAGCTTAGTCGCTGTTCTCAATAAGCTCGCTGATGCTGTGGCAAAAATCGCGGATAAGATG**TAG**

At4g31620 (B3) 1479bp Template: cDNA. Clone: U18158. The protein will be 2 aminoacids longer. (**HQ322384**)

**ATG**GCGAATCATCCACTATCTTCTTCTCGATCCAACCGACCTTTTTTCGTCAGATCACTTGCTGGACACACCTCAAATCTAATAATTCCTGATGAGTTTTTCACTGCTCACTTAGAGGGTAAAACTGGCTTAACGAAATTGAAACTGACTTCTGATGCTTCGGATAGAATCTGGGATGTGAGACTGAACGGCCGGAGATTCGCCGGCGGCTGGGATGATTTCTCCGCCGCACACTGTCTTCGAGACGACGACGTTTTGGTTTTCAGACTCGACGTGAAGATGGTTTTCCACGTCACACCTTCCGGACGCAGTTTCTCACAGATACGTACATCTTCTAGCTCCGGCGACTACGACAGTGATGATGATGATGATGAAGCAGGGGACGATGATTCAGATTCGAAGAACATTTCGTTGAAGAAGAAATCAAGATTTGAAGCAGAGTCTTCTTCATCGGAAAAATATTGTCTTCTCGGCCTCACTGCTTCCAACCTACGCCTAAATCGAGTGAGTTTTACAAAACATTTTTCGAGAGCAAATGGTTTGACGAAGAGATGTTGTATGATTGATCTAATGAATCTAAGTGGGGAATCATGGACTCTGGGTCTGCGACACAACAAAAGAACTGGTCAGGCTTTTATCCGTGGACGCTGGAGAAGTTTCTGCCATGCAAATGAGCTGAAACCCGGATCGTTCTACCGGTTTAAGCTTGTCCGGAATGGGACAAGGCCTTTGCTACAATTGTGTTTTAAAGTTATTCCACAAGGAAACTGTTCCAATTCCAAAGCAAATGGAAAAGCTAATGTTTCTGAAAAGTATAGCAGGGAAGATGGGAGTGCTTCAACGAAGCAGAACAAGTTTCTGACAGTAACCTTGAAGCATTACATGATCCAGTCAGGGCAACTTCGTCTCCGGAGATCATTTGTGAGGGAGAACGGGATCAAGGAGGCTGAAGAGATAATTCTAGTGGACAAAAATGGAGTAGAGTGGCCATCCTATGTATCTTCTTCCAAACAACGAAGAGAGTTTTACATGGCACATGGTTGGATTAGGTTTTGTGAAGCCAATAAGTTAAAGACGGGAGAGACCTTCACGTTGGAGTTTGTTCGAGGAGAAGGCACAACTCCTATGCTCAAGTTCTGCTCCGAGGCCAAG**GTCAAG**ATAGAGCAAGAAGAGGCTCCAGAAGAGAGGGGGACTCCGTTACCAAAGAGAGCTCGAGTGTCTGCAGAAGTAGGACACTCTCGTCGCACTCAGGCACCAAACAAATCCAGTGACGATCCAAAAATCTTGCAGCGCAAGCAACCACTTCAACCCTGCTCATTCTCTGATCAAGCGAAAAAGGTGAAACAGAGTATTGTAAACATTCTAACTGGTATAAAACGGTTTCGGTCAGAGCTTGAGTTAAAGGAGCGGAATTTAGAAGCTGCGCTGCTGGAAATCGACGCCTTAGGGGATAAAGTATCGGAAATCAACAAAATCCTCAAG**TAA**

At5g49230 (ZZ) 636bp Template: cDNA. Clone: U12351. The protein will be 5 aminoacids shorter. (**HQ322385**)

**ATG**GATTCGAATTCATGGATCAATTGTCCCCCTGTGTTTTCATCATCGCCTTCTTCACGCCGTTACCAATCTCGATCAGATTTGTATTTGGGAGATGTTGAAGGAGAAGACGATTTGAAAGCTGAGTTTATGTGTCCTTTTTGTGCAGATGAGTTTGATATTGTTGGTCTTTGTTGTCATATTGATGTGAATCATCCTGTTGAAGCTAAGAATGGGGTTTGTCCTGTCTGCACAAAGAAGGTGGGATTGGACATTGTTGGCCATATAACAACGCAACACGGGAAC**AGATTTTACGTGCAG**CGAAGAAGAAGGTTGCGTAAAGGTGGATATAGCTCTACCTATCTTACTCTTAAAAAAGAGCTTCGAGAAGCTAACTTACAGTCTCTTGGAGGGTCTTCCACTTTCATTCCCTCTTCCAATATAGATTCTGATCCTCTACTCTCATCCTTTATGTTTAAACCCCCTTCGGCTATACCTATCACTGAAGGAGACTCGGTAGCACAAGTCTCACCTAAGGACACCTCTAAAAGCAAAATACAACAAGAATCATTCTCAAACGAAGATCAAGAGAAGGCAAAGAAGAGCAAGTTTGTGCGGGGCTTGCTTTGGTCAACCATGCTTGAAGACAAGTTC**TAA**

At5g67580 (MYB) 900bp Template: cDNA. Clone: U12888. The protein will be 190 aminoacids instead of 299 since it contains intron and a premature stop codon. (**HQ322386**)

**ATG**GGTGCACCAAAGCAGAAGTGGACACCGGAAGAAGAAGCAGCTCTTAAAGCTGGAGTTCTTAAGCATGGGACTGGGAAGTGGCGTACCATACTCTCGGATACTGAGTTTAGTTTAATCCTTAAGTCTCGCTCTAATGTTGATCTTAAGGACAAATGGAGAAATATAAGCGTGACAGCTTTATGGGGTTCAAGGAAGAAGGCTAAACTTGCGCTTAAGAGGACTCCACCAGGTACTAAACAGGATGATAACAACACAGCTCTTACCATTGTGGCTTTGACTAATGATGATGAACGTGCAAAACCAACTTCCCCCGGAGGTTCTGGTGGTGGATCACCACGCACTTGTGCTTCTAAGAGATCAATTACAAGTTTGGATAAGATCATCTTTGAGGCAATTACCAACTTGAGGGAACTACGCGGTTCTGACAGAACATCAATCTTTCTGTATATAGAGGAGAATTTCAAGACTCCACCGAATATGAAAAGGCATGTCGCAGTACGATTAAAGCATTTATCATCGAATGGAACATTAGTTAAGGTAAACACACAACTTTTGATTCAAGATTCA**TGA**TCACTAACACTTCATTACTCTCAGAATTTTCAAAAATACTTACTTGGTGATTTCTCACTTTCAGATAAAGCACAAGTACAGGTTTTCTTCTAATTTTATTCCCGCAGGTGCAAGACAGAAGGCTCCTCAACTCTTTCTGGAAGGAAACAACAAGAAAGACCCAACAAAACCCGAGGAGAACGGTGCCAACAGTCTCACTAAATTCCGAGTAGACGGTGAATTATATATGATAAAAGGCATGACAGCTCAAGAAGCTGCAGAGGCTGCAGCAAGAGCAGTTGCAGAGGCAGAATTTGCTATCACAGAGGCTGAACAAGCAGCAAAAGAGGCAGAAAGAGCAGAAGCAGAAGCTGAAGCTGCTCAGATATTTGCAAAGGCAGCCATGAAAGCTTTGAAGTTCAGGATCCGTAATCATCCTTGGTGA
